# Tissue Non-Specific Genes and Pathways Associated with Diabetes: An Expression Meta-Analysis

**DOI:** 10.3390/genes8010044

**Published:** 2017-01-21

**Authors:** Hao Mei, Lianna Li, Shijian Liu, Fan Jiang, Michael Griswold, Thomas Mosley

**Affiliations:** 1 Department of Data Science, School of Population Health, University of Mississippi Medical Center, Jackson, MS 39216, USA; mgriswold@umc.edu; 2 Shanghai Children’s Medical Center, School of Public Health and School of Medicine, Shanghai Jiaotong University, Shanghai 200127, China; jiangfan@scmc.com.cn; 3 Department of Biology, Tougaloo College, Jackson, MI 39216, USA; lli@tougaloo.edu; 4 Department of Neurology, University of Mississippi Medical Center, Jackson, MS 39216, USA; tmosley@umc.edu

**Keywords:** gene expression, pathway, diabetes

## Abstract

We performed expression studies to identify tissue non-specific genes and pathways of diabetes by meta-analysis. We searched curated datasets of the Gene Expression Omnibus (GEO) database and identified 13 and five expression studies of diabetes and insulin responses at various tissues, respectively. We tested differential gene expression by empirical Bayes-based linear method and investigated gene set expression association by knowledge-based enrichment analysis. Meta-analysis by different methods was applied to identify tissue non-specific genes and gene sets. We also proposed pathway mapping analysis to infer functions of the identified gene sets, and correlation and independent analysis to evaluate expression association profile of genes and gene sets between studies and tissues. Our analysis showed that *PGRMC1* and *HADH* genes were significant over diabetes studies, while *IRS1* and *MPST* genes were significant over insulin response studies, and joint analysis showed that HADH and MPST genes were significant over all combined data sets. The pathway analysis identified six significant gene sets over all studies. The KEGG pathway mapping indicated that the significant gene sets are related to diabetes pathogenesis. The results also presented that 12.8% and 59.0% pairwise studies had significantly correlated expression association for genes and gene sets, respectively; moreover, 12.8% pairwise studies had independent expression association for genes, but no studies were observed significantly different for expression association of gene sets. Our analysis indicated that there are both tissue specific and non-specific genes and pathways associated with diabetes pathogenesis. Compared to the gene expression, pathway association tends to be tissue non-specific, and a common pathway influencing diabetes development is activated through different genes at different tissues.

## 1. Introduction

Diabetes is a chronic metabolic disease of hyperglycemia resulting from defects in insulin secretion, action, or both: the type I diabetes (T1D) is mainly caused by beta-cell destruction and the type II diabetes (T2D) is characterized by defects in insulin action and/or secretion. In the T1D, the cell destruction will eventually eliminate insulin production and lead to absolute insulin deficiency [1]. In contrast, people with the T2D are often resistant to the insulin action. A good understanding of genetics underlying diabetes pathogenesis will play an important role in developing effective prevention, diagnosis and therapy strategy to manage diabetes and relieve its public heath burden.

Diabetes progress and impaired insulin action are accompanied with pathochanges at multiple tissues, including the pancreas, skeletal muscles, the liver and adipose. Of these tissues, the pancreas plays a central role in the diabetes development, and either destruction of its beta cells or reduction of insulin production will lead to the impaired glucose homeostasis. Insulin resistance is a major predictor of T2D and plays an important role in diabetes pathogenesis [2]. More than 80% of insulin-stimulated glucose uptake occurs in skeletal muscle and ~5%–10% of the uptake happens in the adipose tissue [3]. Impaired insulin responses at these tissues will cause abnormal glucose metabolism and the followed hyperglycemia.

In the skeletal muscle, diabetic myotubes is often accompanied with mitochondrial dysfunction, presenting decreased rates of mitochondrial ATP production and substrate oxidation [4]. Hyperglycemia in diabetic patients increases the production of superoxide, resulting in the endothelial dysfunction and decreased numbers of endothelial progenitor cells (EPCs), and diabetes and the impaired progenitor cells are considered to have common pathogenesis [5]. Diabetes progression is associated with arterial pathology, including extracellular matrix changes and increased stiffness in the nonatherosclerotic arterial tissue [6,7]. The liver is also a major tissue taking important roles in glucose homeostasis. Hepatic lipid accumulation is associated with insulin resistance, and the liver can produce various secretory proteins, termed hepatokines, associated with insulin resistance and clinical manifestations of diabetes [8,9]. Pathogenetic study of these tissues contributes to understanding the etiology of impaired insulin action and diabetes development. Although the T1D and T2D have different etiology with pathochanges at multiple tissues, they present some common clinical manifestations and the gene expression study showed that both types of diabetes share pathogenic mechanisms [10].

Advances of high-throughput technology have led to an explosion of gene expression data collected from different tissues in the past decade. The Gene Expression Omnibus (GEO) database has served as a public repository to archive these expression measures, which are generated mostly by microarray technology, and to facilitate retrieval and mining of published expression data [11]. The continuous increase of archived GEO data offers the opportunity to pool gene expression from different studies and tissues, which will help to improve identification of gene signatures associated with a disease that may lack sufficient evidence in a single study before. For this study, we hypothesize that those tissues involved in diabetes pathogenesis share common genetic regulations, and aim to identify tissue non-specific genes and pathways based on expression datasets from the GEO by meta-analysis.

## 2. Materials and Methods

### 2.1. Gene Expression Datasets

We searched the GEO database for gene expression datasets that are related to diabetes and insulin response. Our analysis is focused only on those manually curated GEO datasets (GDSs) that were directly downloaded and parsed through R package of GEO query [12]. The expression level of every gene, measured as *M* value (i.e., log 2-expression level), was extracted for follow-up analysis. GDSs will be merged to a single study if the datasets are expression measures from the same samples but different microarray platforms. Gene expression of every study is measured as the largest *M* value if the gene was assayed on multiple platforms [13]. To make gene expression comparable across samples, all probe *M* values were normalized by quantile normalization from the *R* package, preprocessCore [14,15].

### 2.2. Gene Expression Association Test and Meta-Analysis

The empirical Bayes-based linear regression method [16] was applied to test differential gene expression based on the null hypothesis that expression of *M* value is equal across all *K* phenotypes: *M*_1_ *= M*_2_ *= … = M_i_ … = M_k_* (*K* ≥ 2), where *M_i_* can be the case status of T1D and T2D, healthy control, insulin resistant or insulin sensitive. A significant test will suggest gene association with diabetes and insulin response. The analysis was performed by the *R* package, limma [16], and the standard errors of tests were moderated across genes by empirical Bayes model to calculate *F* statistic and *p*-value for every gene. The *U*-score [17] of the *i*-th gene ($U_{i}$) is calculated as $U_{i}=\left(\sum_{j}I\left(p_{j}<p_{i}\right)+0.5\cdot \sum_{j}I\left(p_{j}=p_{i}\right)\right)/N$, where *p_i_* is *p*-value of the *i*-th gene and *N* is the total number of measured genes. The *U*-score approximately follows uniform distribution, estimating the percentage of genes with stronger expression association than the tested one. We hypothesize that 5% of genes are associated with diabetes, and a gene with *U*-score ≤ 0.05 is defined as significant for following pathway test and meta-analysis.

Meta-analysis was conducted by the binomial test to evaluate differential gene expressions over studies and tissues. The binomial test counted the number of significant genes with *U*-score *≤* 0.05 over studies as a random variable *X*, which follows a binomial distribution with probability of 0.05, and the meta-analysis *p*-value was calculated as $Bin\_P=Pr\left(X\geq \sum_{i=1}^{M}I\left(U_{i}\leq 0.05\right)\right)$. The significance of Bin_P is based on the Bonferroni adjustment for the total number of genes.

### 2.3. Pathway Expression Association Test and Meta-Analysis

Pathway expression association was examined by testing enrichment of knowledge-based gene sets for significant genes. The test was based on the MSigDB knowledge base [18] that contains curated information of over 10,000 gene sets extracted from different public pathway databases, e.g., the Kyoto Encyclopedia of Genes and Genomes (KEGG) [19]. The enrichment analysis was conducted by the hypergeometric test of significant genes with *U*-score ≤ 0.05 using the *R* package *snpGeneSets* [17,20]. The pathway effect was estimated as the proportion of significant genes in the gene set minus 5%. The pathway *p*-value (path_p) was calculated based on hypergeometric distribution, and the adjusted *p*-value ($path\_p_{a}$) was obtained by a permutation test to adjust for multiple testing.

Meta-analysis was conducted by the fixed-effect model and the binomial test to measure pathway expression associations across studies. The fixed-effect model with inverse of variance as study-specific weight was applied to estimate pathway enrichment effect over all studies, and meta-analysis *p*-value (Fixed_p) was calculated to test the null hypothesis of effect = 0. The analysis was performed by the *R* package of metaphor [21]. The binomial test calculates meta-analysis *p*-value based on unadjusted (path_p) and adjusted pathway *p*-value ($path\_p_{a}$), respectively, over *M* studies as $Bin\_p0=Pr\left(X\geq \sum_{i=1}^{M}I\left({\left(path\_p\right)}_{i}\leq 0.05\right)\right)$ and $Bin\_p1=Pr\left(X\geq \sum_{i=1}^{M}I\left({\left(path\_p_{a}\right)}_{i}\leq 0.05\right)\right)$, where X is a random variable following binomial distribution with size *M* and probability of 0.05. The significance of Fixed_p and Bin_p0 is based on the Bonferroni adjustment for the number of tested gene sets, while Bin_p1  is significant if the value is ≤0.05.

### 2.4. KEGG Pathway Mapping Analysis

The KEGG [22] pathway database describes manually curated molecular interaction and reaction networks, and provides pathway maps for common human diseases. The mapping analysis, similar to the enrichment analysis above, applied hypergeometric test by the *R* package *snpGeneSets* to examine if the MSigDB gene set significantly overlaps a KEGG pathway [20]. The mapping effect estimates the higher probability for a gene of the MSigDB gene set than a random gene, while they also belong to the KEGG pathway [20]. The mapping *p*-value is based on the hypergeometric distribution and the adjusted *p*-value is obtained by 10,000 permutation tests. A significant test with adjust *p*-value ≤ 0.05 suggests that the KEGG pathway is correlated with the MSigDB gene set, and they potentially share common functions.

### 2.5. Correlation and Independent Analysis of Expression Association Profile between Studies

To investigate the profile of expression association with diabetes and insulin response between studies, we conducted correlation and independent analysis for both genes and MSigDB gene sets. The correlation analysis was based on Spearman’s rank correlation test by the *R* function of *cor.test*, which aimed to examine similar expression association profiles between studies. The independent analysis was based on the *U*-score of gene set association and tested by the McNemar’s method with the *R* function of *mcnemar.test*. The pathway *U*-score was calculated the same as the gene *U*-score above, and the value ≤ 0.05 indicated that its association strength ranked at the top 5%. The independent analysis counted the inconsistent number of genes and gene sets with *U*-score ≤ 0.05 at one study but *U*-score > 0.05 at the compared study, which aimed to examine different expression association between studies. For both types of analyses, the Bonferroni method was applied to adjust for multiple testing.

## 3. Results

### 3.1. Characteristics of the Gene Expression Datasets and Studies

Our search against the GEO database formed 13 gene expression studies based on 14 GDSs of diabetes states, including T1D and T2D, and the tissues include skeletal muscles, myotube, pancreas, liver, blood cells, endothelial progenitor cells (EPCs), arteries and adipose. The search also generated five expression studies based on 11 GDSs of insulin actions, and the tissues include skeletal muscles and adipose. The characteristics of all expression studies were summarized at the Table 1, showing the study ID, the GDS ID, the microarray platform, the PUBMED ID, the number of genes measured, the sample size, the contrast test for differential gene expression and the tissue.

### 3.2. Tissue Non-Specific Gene Expression Association

We performed differential expression tests for 6889 and 7332 genes that were measured at all studies of diabetes and insulin actions, respectively, and the corresponding adjusted significance levels by Bonferroni correction were 7.26E−06 and 6.82E−6. The meta-analysis showed that the genes of progesterone receptor membrane component 1 (*PGRMC1*) and hydroxyacyl-CoA dehydrogenase (*HADH*) were significant over 13 studies of diabetes with *p*-values (*Bin_P*) of 1.03E−6 and 1.03E−6 respectively, and the genes of insulin receptor substrate 1 (*IRS1*) and mercaptopyruvate sulfurtransferase (*MPST*) were significant across five studies of insulin action with *Bin_P* of 3.13E−7. *U*-scores and *p*-values of the four genes at every study were summarized in Table 2 and the gene descriptions were shown in Supplementary Table S1.

The meta-analysis results (Table 2) showed that the *PGRMC1* were significant across diabetes studies, presenting six out of 13 studies with the *U*-score ≤ 0.05, and the *IRS1* gene was significant in four studies of insulin response (i.e., *U*-score ≤ 0.05). The joint analysis of all 17 studies showed that the *HADH* and the *MPST* were significant with *p*-values of 6.31E−7 and 6.28E−8, respectively. The *HADH* was significant in six diabetes studies (studies 1, 3, 5, 8, 11 and 13) of adipose, blood, EPC, myotube, pancreas and skeletal muscles, and the gene had the smallest *U*-score of 0.44% at the study 11 of pancreas that compared T2D with non-diabetes. Of the five studies of insulin response, the *HADH* had the *U*-score of 3.71% in study 5 of adipose. The *MPST* was significant in four insulin response studies of skeletal muscles with the smallest *U*-score of 0.86% (study 4), and the significant differential expression was also observed in four diabetes studies of adipose, arteries, blood and the liver (study 1, 2, 4 and 7) with the smallest *U*-score of 0.20% (study 4).

### 3.3. Tissue Non-Specific Pathway Expression Association

We performed meta-analysis of the pathway expression test for MSigDB gene sets over 13 diabetes studies, five insulin action studies and their combined data sets. The meta-analysis *p*-values of *Fixed_p* and *Bin_p0* used significant levels of 5.0 × 10^−6^ based on the Bonferroni correction for about 10,000 gene sets, while the *p*-value of *Bin_p1* directly took the significant level of 0.05, due to fact that its calculation was based on adjusted pathway *p*-values of individual expression studies. The analysis identified six significant gene sets at the diabetes studies and the combined datasets, including “UV response”, “chronic myelogenous leukemia”, “KLF1 targets”, “SMARCA2 targets”, “Alzheimer’s disease” and “stromal stem cells”. *p*-values of the six gene sets by different methods were shown in Table 3, and a description of these gene sets can be found at the Supplementary Table S2. The detailed results for every study were summarized at the Supplementary Tables S3–S8 with the forest plots shown at the Supplementary Figures S1–S6.

Meta-analysis of diabetes studies showed that the *Fixed_p* and *Bin_p0* of the six gene sets ranged at 1.45 × 10^−38^–1.88 × 10^−15^ and 3.47 × 10^−13^–4.01 × 10^−08^, respectively, while the *Bin_p1* ranged at 3.10 × 10^−3^~1.97 × 10^−5^ (Table 3). The gene sets were also consistently confirmed at the meta-analysis of all combined data sets: the “chronic myelogenous leukemia” had the smallest *p*-values of *Fixed_p* (3.91 × 10^−44^) and *Bin_p1* (1.52 × 10^−5^) and the second smallest *p*-value of *Bin_p0* (3.41 × 10^−12^); and the “Alzheimer’s disease” had the least significant *p*-values of *Fixed_p* (1.84 × 10^−18^), Bin_*p0* (2.95 × 10^−9^) and *Bin_p1* (1.55 × 10^−3^). The pathway enrichment analyses showed that the “chronic myelogenous leukemia” had adjusted *p*-value < 0.05 in five diabetes expression studies of adipose, arteries, blood and pancreatic tissues with effect = 2.87%–7.07% and the insulin response study of skeletal muscles with effect *=* 4.63% (Supplementary Table S4); the “Alzheimer’s disease” had adjusted *p*-value < 0.05 in four diabetes studies of arteries, blood and pancreatic tissues with effect *=* 3.10%–4.06% (Supplementary Table S7). The joint analysis also showed that the *p*-values of *Fixed_p*, *Bin_p0* and *Bin_p1* were 9.46E−32, 1.12E−10 and 1.55E−03 for “UV response”, 4.72E−29, 1.54E−15 and 1.55E−03 for “KLF1 targets”, 1.11E−27, 6.28E−08 and 1.55E−03 for “SMARCA2 targets”, and 2.93E−22, 1.12E−10 and 1.72E−04 for “stromal stem cells”.

The six significant gene sets were mainly observed in diabetes studies 1, 2, 3, 4, 8, 10 and 11 and insulin studies 3 and 4 (adjusted *p*-value < 0.05), involving tissues of adipose, arteries, blood, myotube, pancreatic tissues and skeletal muscles (Supplementary Tables S3–S8 and the Supplementary Figures S1–S6). Specifically, all six gene sets were significant at the diabetes study 3 of blood tissue (GDS3874/GDS3875); five gene sets except the “stromal stem cells” were significant in the diabetes study 2 of artery tissue (GDS3980); four gene sets except the “UV response” and the “stromal stem cells” were significant at the diabetes study 10 of pancreas (GDS3882); four gene sets except the “Alzheimer’s disease” and the “stromal stem cells” were significant in the insulin study 4 of skeletal muscles (GDS3715); diabetes study 8 of myotube (GDS3681) and insulin study 3 of skeletal muscles (GDS3181) contained only a significant gene set of “stromal stem cells”; and diabetes study 4 of blood (GDS3963) had only a significant gene set of “chronic myelogenous leukemia”.

### 3.4. Mapped KEGG Pathways for the Identified Gene Sets

To infer the potential functions of significant gene sets, mapping analysis was conducted to find their related KEGG pathways by mapping analysis. The most related KEGG pathways with estimated effects were shown at the Table 4. The “UV response” was mapped to the transforming growth factor beta 1 (TGF-beta) signaling pathway, presenting the effect of 20%, standard error (SE) of 0.03, *p*-value of 1.28E−9 and permutation-adjusted *p*-value < 0.0001. Similarly, we identified that the “chronic myelogenous leukemia”, “KLF1 targets” and “SMARCA2 targets” were mapped to citrate cycle pathway (effect = 35% and adjusted *p*-value < 0.01), DNA replication (effect = 24% and adjusted *p*-value = 0.016) and nucleotide excision repair (effect = 11% and adjusted *p*-value = 0.034), respectively. The gene set of “Alzheimer’s disease” was mapped to the pathways of “Oxidative phosphorylation” and “Parkinson’s disease” with an effect of 35% and adjusted *p*-value < 0.001; and the “stromal stem cells” was mapped to the peroxisome proliferator-activated receptors (PPAR) and p53 signaling pathways with an effect of 10% and adjusted *p*-value of 0.029.

### 3.5. Correlation and Independence of Gene and Pathway Expression Associations

**Figure 1:**
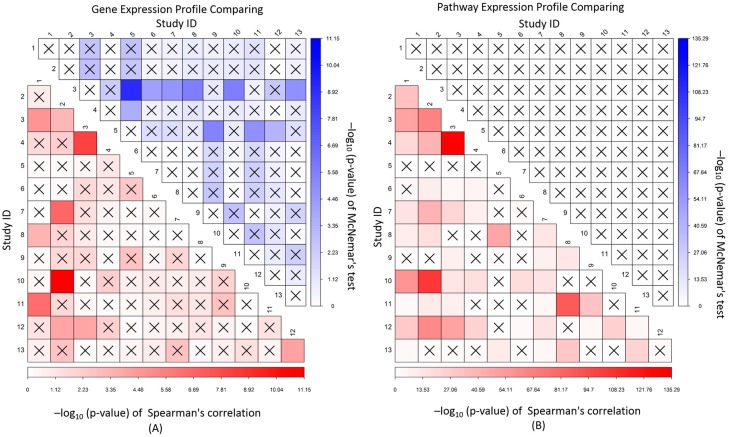
Correlation and independent analyses of gene (**A**) and pathway (**B**) expression association profiles between studies. The lower triangle is the correlation analysis and the upper triangle is the independent test. The ‘X’ indicates the analysis *p*-value is not significant.

Correlation and independent analyses of expression association profiles were conducted among the 13 diabetes studies, and a plot of the results was shown at the Figure 1. After adjustment for multiple testing, significantly correlated and independent association profiles of gene expression, respectively, accounted for 10 out of 78 analyses (or 12.8%) between studies (Supplementary Tables S9 and S10). However, for pathway expression, no studies were to be observed with significantly independent association, and, in contrast, 46 analyses (59.0%) were found to have significant correlation (Supplementary Table S11). The results showed that studies with correlated gene association profiles also tended to have correlated pathway association profiles. Study 2 of arteries and study 10 of the pancreas had the strongest gene correlation (*ρ* = 0.074 and *p*-value = 7.07E−12) and the second strongest pathway correlation (*ρ* = 0.21 and *p*-value = 9.20E−104), while studies 3 and 4 of blood had the second strongest gene correlation (*ρ* = 0.065 and *p*-value = 6.65E−9) and the strongest pathway correlation (*ρ* = 0.24 and *p*-value = 5.17E−136). The results suggested that different studies have both tissue specific and non-specific gene expression association with diabetes, and compared to the gene expression, the pathway expression tends to be tissue non-specific. Analyses of the insulin studies presented consistent conclusions.

## 4. Discussion

Although diabetes may have different etiology, they usually present some common clinical manifestations and share pathogenic mechanisms that are accompanied with pathochanges at different tissues. There was evidence in the animal studies showing that different tissues involve common genetic regulations in diabetes development. For example, GLUT4 heterozygous knockout in mice exhibited decreased expression in adipose tissue and muscle [35], and the knockouts in different tissues led to common observations of impaired whole-body glucose homeostasis and developed insulin resistance [36,37]. For this meta-analysis, we hypothesize that there exist tissue non-specific genetic regulations influencing human diabetes pathogenesis, and the study aim is to identify these genes and pathways based on measured gene expressions. Comparing to the original expression data submitted by the researcher, which is heterogeneous and may not be directly analyzed, our study is focused on the GEO Datasets that are curated by the NCBI and consisted of biologically and statistically comparable data [11].

We searched the GEO database and identified 27 gene expression datasets from different tissues, of which 14 datasets were related to 13 expression studies of diabetes states and 11 datasets were linked to five expression studies of insulin responses. The gene expressions were measured from different tissues including pancreas, skeletal muscles, liver, adipose and blood. For every study, we analyzed differential gene expressions to test gene association by an empirical Bayes approach that has robust behavior even for small sample size [16], and examined pathway expression association by hypergeometric test for enrichment of significant genes that provides parametric estimate of effect and calculation of *p*-value. We performed meta-analysis of measured genes and MSigDB gene sets over studies for identification of tissue non-specific genes and pathways. Our meta-analysis strategy consisted of tests for both genes and pathways. For pathway meta-analysis, two types of tests were also conducted to provide consistent evaluation of expression association: the binomial test was based on the number of significant studies, and the fixed-effect model was based on the sum of effects over studies.

Our meta-analysis showed that *PGRMC1*, *HADH*, *IRS1* and *MPST* were the four tissue non-specific genes presenting differential expression association with the diabetes or insulin response. These four genes are expressed in most tissues. For *PGRMC1* and *HADH*, their associations ranked at the top 5% (i.e., *U*-score *≤* 0.05) in the six diabetes studies and their best *U*-scores were 0.34% and 0.44% in the diabetes studies 9 and 11 of the pancreas, respectively (Table 2). Previous studies have indicated they are both related to the insulin secretion. The *PGRMC1* is located at the Chr X, encoding a progesterone steroid receptor. It interacts the glucagon-like peptide-1 (GLP-1) receptor, and its overexpression enhances GLP-1-induced insulin secretion [38]. The *HADH* is mapped to the Chr 4q22–26. It encodes an enzyme, which is crucial for β-oxidation of fatty acids and generation of acetyl-CoA and associated with ketogenesis. Downregulation of HADH mRNA and the gene mutations are associated with insulin secretion and hyperinsulinaemic hypoglycaemia [39].

The *IRS1*, located at the Chr 2q36, encodes a protein phosphorylated by insulin receptor tyrosine kinase, which is required for hormonal control of metabolism. The IRS1 protein is critical for insulin response, and impairment of insulin signaling by IRS1 is linked to insulin resistance [40]. The *MPST* gene, mapped to the Chr 22q13.1, encodes the 3-mercaptopyruvate sulfurtransferase. The enzyme is known to produce the hydrogen sulfide (H_2_S) from cysteine and the increased H_2_S in adipose tissues was observed to inhibit insulin-stimulated glucose metabolism and regulate insulin sensitivity [41]. Both IRS1 and MPST had *U*-score ≤ 0.05 in four out of five insulin studies. The IRS1 also showed a small *U*-score of 0.003% in the diabetes study 4 of blood. The MPST presented *U*-score ≤ 0.05 in four diabetes studies with the best *U*-score of 0.2% from study 4 of blood. The results suggested that both genes are related to insulin response, and their abnormal expression levels in the blood indicate the progression of diabetes.

Our pathway meta-analysis has identified six MSigDB gene sets with significant expression associations. However, genetic mechanisms of these gene sets and their biological functions related to diabetes remain unknown. We therefore proposed the mapping analysis and aimed to infer their roles underlying diabetes pathogenesis by the most related KEGG pathways. The six gene sets were significant in diabetes studies and joint analysis by all three *p*-values (*Fixed_p*, *Bin_p0* and *Bin_p1* at the Table 3): (1) the gene set of “UV response” are genes downregulated in fibroblasts after UV irradiation, and it was mapped to the TGF-beta signaling pathway, which regulates insulin gene transcription and β cell function [42]; (2) the gene set of “chronic myelogenous leukemia” (CML) is a collection of genes upregulated in the CD34+ cells of CML patients and previous study suggested that CML is connected to T2D [43]. The gene set was mapped to the pathway of the citrate cycle that is related to glucose metabolism and diabetes progression [44]; (3) the “KLF1 targets” is a collection of genes discovered to be downregulated in erythroid progenitor cells due to knockout of KLF1 gene, and the mapping study showed that it was related to the DNA replication pathway, suggesting its effects on diabetes potentially through pancreatic β-cell replication [45]; (4) the “SMARCA2 targets” presents genes positively correlated with the SMARCA2 gene, and its mapped pathway of nucleotide excision repair (NER) is responsible for recognizing and repairing bulky DNA damage that is commonly observed in diabetic patients [46]; (5) the “Alzheimer’s disease” (AD) gene set lists genes that are downregulated in the brains of Alzheimer’s patients, and it is mapped to pathways of oxidative phosphorylation that have important roles in causing diabetes [47] and Parkinson’s disease that are known to have shared mechanisms with diabetes as AD [48]; and (6) the “stromal stem cells” gene set is identified as a group of genes upregulated in cultured stromal stem cells from adipose tissue, and the mapped PPAR and p53 signaling pathways are associated with insulin sensitivity [49] and insulin resistance [50], respectively.

To further evaluate the identified tissue non-specific genes and gene sets, we proposed the correlation and independent analyses for their expression association profile between different studies and tissues. The results showed that correlated association gene profiles accounted for 12.8% analyses: for example, diabetes study 2 of artery tissue and study 10 of the pancreas had rank correlation *p*-value of 7.07E−12, and study 1 of adipose tissue and study 11 of pancreas had rank correlation *p*-value of 5.59E−7 (Supplementary Table S9). The results also showed that 12.8% analyses had significantly independent gene association profile: for example, study 3 of EPC and study 5 of blood had the *p*-value of 7.47E−10. However, most studies did not present obviously correlated or independent profiles of gene expression association. In contrast, for pathway expression association, no studies showed a significantly independent profile, but 59.0% analyses had significant correlation. The results indicated that most tissues and studies have similar profiles of pathway expression associations with diabetes, and compared to genes, diabetes pathways tend to be tissue non-specific. For example, study 2 of arteries and study 10 of the pancreas had their rank correlation (*p*-value) of expression association profile changed from 0.074 (7.07E−12) for genes to 0.21 (9.20E−104) for pathways; and study 3 of EPC and study 5 of blood had independent *p*-value of expression association profile changed from 7.47E−10 for genes to >0.05 for pathways. The results suggested that a common pathway is mainly activated through tissue specific genes in different tissues to influence diabetes pathogenesis.

Our meta-analysis was performed on curated GDS of gene expressions identified from the GEO. However, these datasets have a few limitations: (1) most diabetes studies are for T2D and only two studies are for T1D; (2) all expression datasets have a relatively small sample size (≤117); and (3) many tissues were collected in only 1–3 studies. These limitations can affect the statistical test and reduce the study power. Based on results of this analysis, it is worthwhile to conduct replication studies on more expression datasets with large sample size and different tissues in the next step. In addition, identification of tissue non-specific genes and pathways in the current study mainly relied on significantly statistical tests, which, however, had the limitation to provide direct evidence for their roles in diabetes pathogenesis. Therefore, in vivo biological studies of these genes and pathways in the future will play essential roles in understanding their genetic regulation mechanisms of diabetes.

## 5. Conclusions

In summary, we examined gene expression datasets from the GEO database that are related to the diabetes and insulin response, and performed meta-analysis with the aim to identify tissue non-specific genes and pathways. We also proposed the KEGG pathway mapping analysis to infer the function of MSigDB gene sets, and correlation and independent analysis of expression association profile between different studies and tissues. Our study successfully identified four and six tissue non-specific genes and gene sets, respectively. The results also suggested that effects of diabetes-related pathways are more likely tissue non-specific, compared to the effects of diabetes genes.

## Figures and Tables

**Table 1:** Characteristics of the gene expression studies.

Study	GDS_ID	GPL_ID	Pub_ID	N_genes	Size	Contrast	Tissue
**Diabetes State**							
1	GDS3665	GPL2986		16,075	10	T2D vs. control	adipose
2	GDS3980	GPL571	21926180 [6]; 22340758 [7]	12,778	21	T2D vs. control	artery
3	GDS3874	GPL96	17595242 [10]	18,552	117	T1D vs. healthy and T2D vs. healthy	blood
	GDS3875	GPL97					
4	GDS3963	GPL6883	21829658 [23]	17,476	24	T2D vs. impaired fasting glucose vs. control	blood
5	GDS3656	GPL2700	19706161 [5]	16,778	32	T1D vs. Healthy	EPC
6	GDS3876	GPL96	19549744 [9]	12,779	18	obese T2D vs. obese no T2D	liver
7	GDS3883	GPL570	21035759 [8]	20,539	17	T2D vs. normal glucose tolerance	liver
8	GDS3681	GPL8300	18719883 [4]	8861	20	T2D vs. control	myotube
9	GDS3782	GPL1352	20644627 [24]	20,185	20	T2D vs. control	pancreas
10	GDS3882	GPL96	21127054 [25]	12,779	13	T2D vs. non-diabetes	pancreas
11	GDS4337	GPL6244	22768844 [26]	17,323	63	T2D vs. non-diabetes	pancreas
12	GDS3880	GPL570	22802091 [27]	20,539	42	T2D vs. pre-diabetes vs. normoglycemic control	skeletal muscle
13	GDS3884	GPL570	21393865 [28]	20,539	50	T2D vs. Normoglycemia with FH+ vs. Normoglycemia with FH−	skeletal muscle
**Insulin Action**							
1	GDS157	GPL80	12436343 [29]	13,742	10	insulin resistant vs. insulin sensitive	skeletal muscle
	GDS158	GPL98					
	GDS160	GPL99					
	GDS161	GPL100					
	GDS162	GPL101					
2	GDS2790	GPL80	17472435 [30]	12,885	12	Before vs. after Hyperinsulinemic-euglycemic clamp for nondiabetes	skeletal muscle
	GDS2791	GPL96					
3	GDS3181	GPL96	18334611 [31]	12,779	36	−60 vs. 30 vs. 240 min of Hyperinsulinemic-euglycemic clamp for nondiabetes	skeletal muscle
4	GDS3715	GPL91	17709892 [32]; 21109598 [33]	8768	110	Diabetes vs. insulin sensitive vs. insulin resistant before and after Hyperinsulinemic-euglycemic clamp	skeletal muscle
5	GDS3781	GPL570	20678967 [34]	20,539	39; 19	insulin sensitive vs. insulin resistant	adipose
	GDS3962						

GDS_ID: the GDS ID of the expression dataset; GPL_ID: ID of the platform for generating the expression dataset; PUB_ID: the publication ID at the PubMed database; N_genes: the number of genes measured for expression level; Size: the number of samples at the study; Contrast: it presented the test of differential gene expression between two or more phenotypes by the regression method; EPC: endothelial progenitor cells; FH+: family history of diabetes; FH-: no family history of diabetes; T1D: type I diabetes; T2D: type II diabetes.

**Table 2:** Differential gene expression and meta-analysis.

		PGRMC1	HADH	IRS1	MPST
**Study**	**GDS_ID**	**Gene *U*-Score (%) of Diabetes State**			
1	GDS3665	***4.79***	***0.48***	9.17	***2.68***
2	GDS3980	31.31	14.46	20.64	***3.64***
3	GDS3874/GDS3875	8.55	***3.48***	24.46	97.48
4	GDS3963	45.03	48.6	***3.01E−03***	***0.2***
5	GDS3656	***0.52***	***3.35***	68.95	89.07
6	GDS3876	***4.16***	82.07	89.31	35.61
7	GDS3883	97.9	69.24	15.34	***3.85***
8	GDS3681	88.77	***2.73***	81.4	27.87
9	GDS3782	***0.34***	7.43	81	79.18
10	GDS3882	***2.33***	66.82	95.75	47.81
11	GDS4337	***4.66***	***0.44***	7.54	37.53
12	GDS3880	95.17	50.5	41.35	6.55
13	GDS3884	96.25	***2.97***	48.08	54.24
	***Bin_P***	1.03E−6	1.03E−6	0.14	2.87E−4
**Study**	**GDS_ID**	**Gene *U*-Score (%) of Insulin Action**			
1	GDS157/GDS158/GDS160/GDS161/GDS162	7.48	NA	***3.82***	***2.2***
2	GDS2790/GDS2791	9.59	10.14	***2.5***	***3.92***
3	GDS3181	55.03	16.78	***3.46***	***4.56***
4	GDS3715	30.9	82.89	25.76	***0.86***
5	GDS3781/GDS3962	48.65	***3.71***	***4.52***	23.59
	***Bin_P***	0.23	0.01	3.13E−7	3.13E−7
**Joint Analysis of Combined Diabetes State and Insulin Action**					
	***Bin_P***		6.31E−7		6.28E−8

The bold italic font indicates significantly differential gene expression (i.e., *U*-score ≤ 5%).

**Table 3:** Meta-analysis *p*-values of significant gene sets.

PID	GeneSet	*Fixed_p*	*Bin_p0*	*Bin_p1*
Diabetes Studies				
5599	UV response	7.72E−17	4.01E−08	3.10E−03
4914	chronic myelogenous leukemia	1.45E−38	1.16E−09	1.97E−05
7922	KLF1 targets	3.35E−26	3.47E−13	3.10E−03
5947	SMARCA2 targets	1.95E−25	4.01E−08	3.10E−03
6442	Alzheimer’s disease	1.65E−19	4.01E−08	2.87E−04
7145	stromal stem cells	1.88E−15	1.16E−09	2.87E−04
Insulin Response Studies				
5599	UV response	1.48E−18	3.00E−05	0.023
4914	chronic myelogenous leukemia	4.27E−08	3.00E−05	0.023
7922	KLF1 targets	4.55E−05	3.00E−05	0.023
5947	SMARCA2 targets	1.12E−04	0.023	0.023
6442	Alzheimer’s disease	0.042	1.16E−03	0.23
7145	stromal stem cells	2.14E−08	1.11E−03	0.023
Joint Analysis				
5599	UV response	9.46E−32	1.12E−10	1.55E−03
4914	chronic myelogenous leukemia	3.91E−44	3.41E−12	1.52E−05
7922	KLF1 targets	4.72E−29	1.54E−15	1.55E−03
5947	SMARCA2 targets	1.11E−27	6.28E−08	1.55E−03
6442	Alzheimer’s disease	1.84E−18	2.95E−09	1.55E−03
7145	stromal stem cells	2.93E−22	1.12E−10	1.72E−04

PID: ID of the significantly identified gene sets; GeneSet: name of the gene sets; *Fixed_p*: the unadjusted *p*-value by fixed-effect meta-analysis; *Bin_p0*: the unadjusted meta-analysis *p*-value by binomial test; *Bin_p1*: the adjusted meta-analysis *p*-value by binomial test.

**Table 4:** The closest KEGG Pathway

Gene Set	KEGG Pathway	Size	Gene	Effect	SE	*p*	adj_p
UV response	TGF-beta signaling pathway	86	22	0.20	0.03	1.28E−09	<0.001
chronic myelogenous leukemia	The citrate cycle	32	15	0.35	0.06	1.11E−06	<0.001
KLF1 targets	DNA replication	36	13	0.24	0.05	1.54E−04	0.016
SMARCA2 targets	Nucleotide excision repair	44	6	0.11	0.02	4.29E−04	0.034
Alzheimer’s disease	1. Oxidative phosphorylation	135	62	0.35	0.03	9.20E−27	<0.001
	2. Parkinson’s disease	133	60	0.35	0.03	2.13E−25	<0.001
stromal stem cells	1. PPAR signaling pathway	69	9	0.10	0.02	3.02E−4	0.029
	2. p53 signaling pathway	69	9	0.10	0.02	3.02E−4	0.029

Size: the number of genes in the KEGG pathway; Gene: the number of overlapped genes between the gene set and the KEGG pathway; Effect: the higher probability for a gene of the gene set that belongs to the KEGG pathway than a random gene; SE: the standard error of the estimated effect; *p*: the unadjusted *p*-value; and adj_p: the adjusted *p*-value by permutation test.

## Supplementary Materials

The following are available online at www.mdpi.com/2073-4425/8/1/44/s1, Figure S1: Forest plot of UV response (DACOSTA_UV_RESPONSE_VIA_ERCC3_DN); Figure S2: Forest plot of chronic myelogenous leukemia (DIAZ_CHRONIC_MEYLOGENOUS_LEUKEMIA_UP). Figure S3: Forest plot of KLF1 targets (PILON_KLF1_TARGETS_DN); Figure S4: Forest plot of SMARCA2 targets (SHEN_SMARCA2_TARGETS_UP); Figure S5: Forest plot of Alzheimer's disease (BLALOCK_ALZHEIMERS_DISEASE_DN); Figure S6: Forest plot of stromal stem cells (BOQUEST_STEM_CELL_CULTURED_VS_FRESH_UP); Table S1: Description of differentially expressed genes; Table S2: Description of significantly identified gene sets; Table S3: Gene set enrichment analysis of UV response; Table S4: Gene set enrichment analysis of chronic myelogenous leukemia; Table S5: Gene set enrichment analysis of KLF1 targets; Table S6: Gene set enrichment analysis of SMARCA2 targets; Table S7: Gene set enrichment analysis of Alzheimer’s disease; Table S8: Gene set enrichment analysis of stromal stem cells; Table S9: Significant correlation of gene expression association; Table S10: Significant independence of gene expression association; Table S11: Significant correlation of pathway expression association. All expression datasets are available at the https://www.ncbi.nlm.nih.gov/geo/ and can be accessed by the GDS IDs.
