# Supplementary material for: Tissue Non-Specific Genes and Pathways Associated with Diabetes: An Expression Meta-Analysis
Source: Genes (Basel). 2017 Jan 21;8(1):44. doi: 10.3390/genes8010044 (PMC5295038; doi:10.3390/genes8010044)
Supplement: Supplementary file 1 [file genes-08-00044-s001.pdf]

Supplementary Materials: Tissue Non-Specific Genes and Pathways Associated with Diabetes: An Expression Meta-Analysis

Hao Mei, Lianna Li, Shijian Liu, Fan Jiang, Michael Griswold and Thomas Mosley

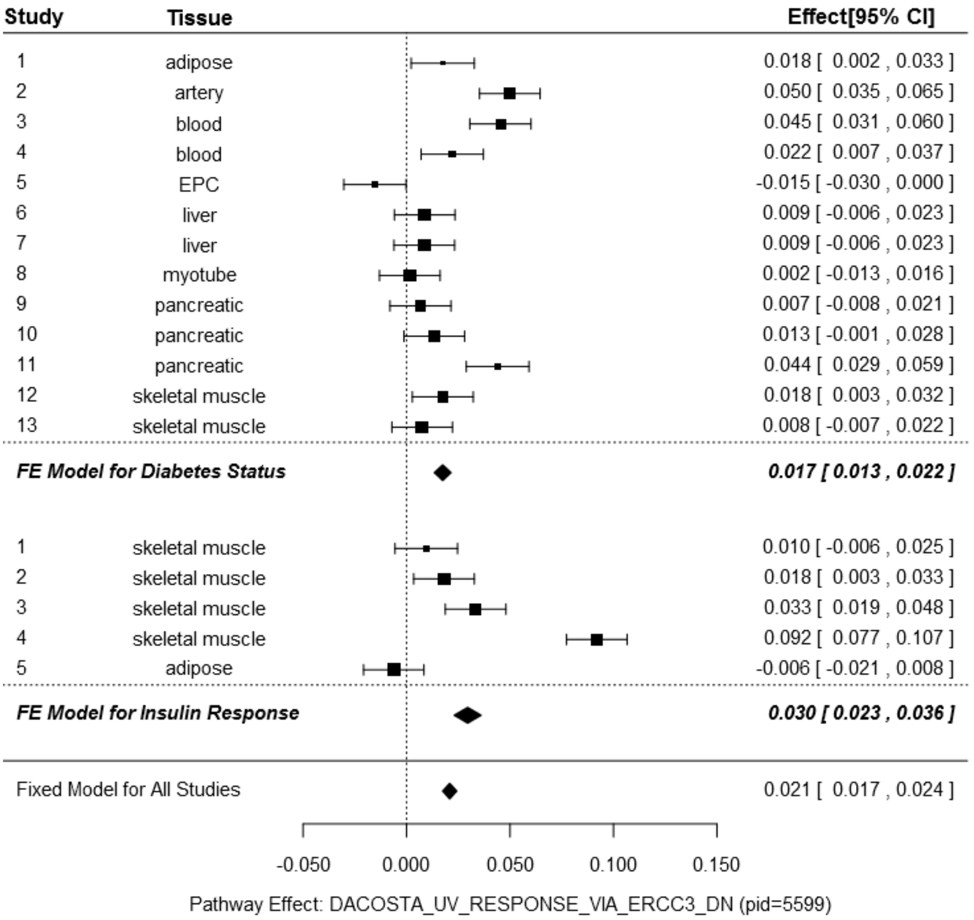

Figure S1. Forest plot of UV response (DACOSTA_UV_RESPONSE_VIA_ERCC3_DN).

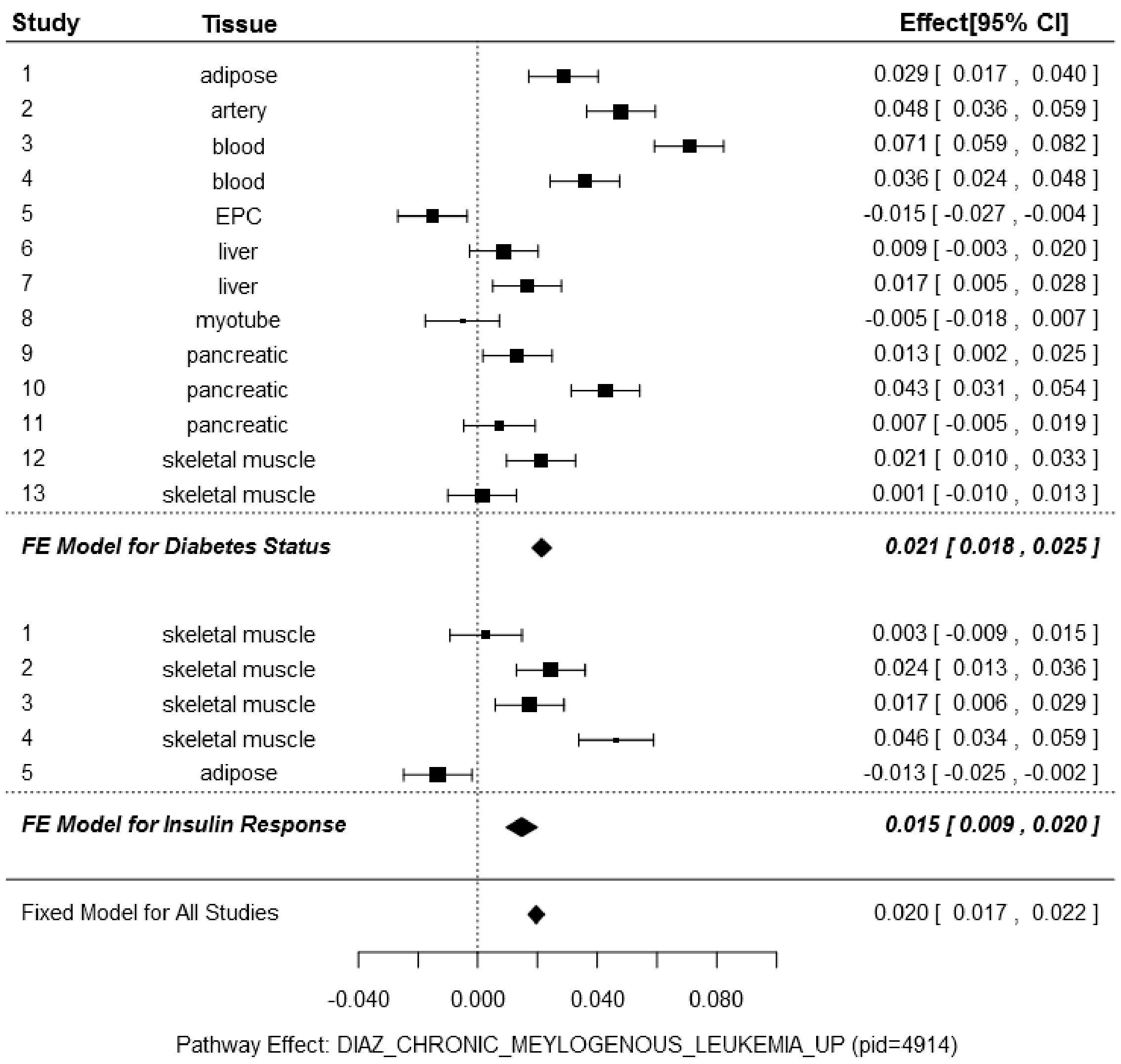

Figure S2. Forest plot of chronic myelogenous leukemia (DIAZ_CHRONIC_MEYLOGENOUS_LEUKEMIA_UP).

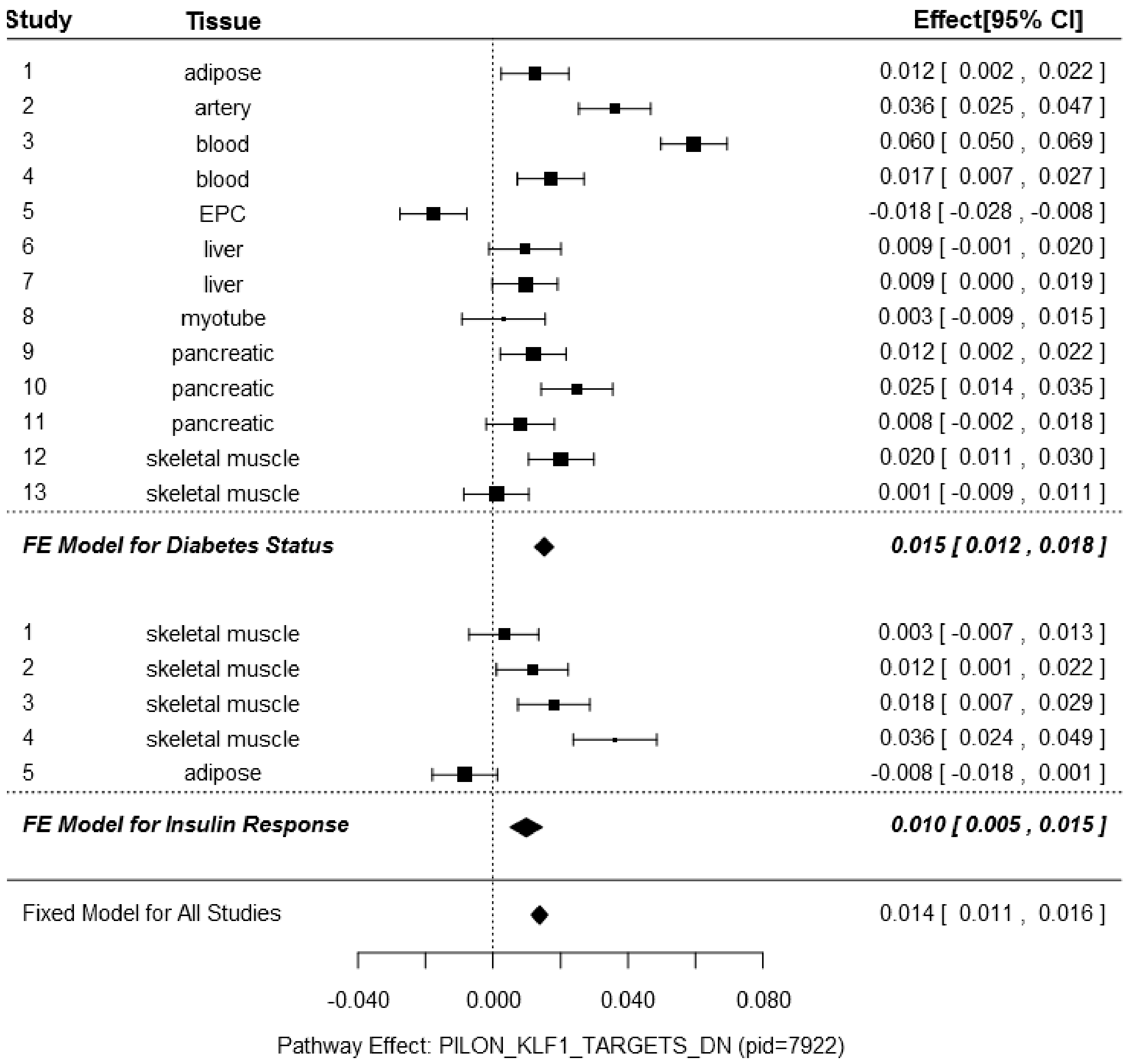

Figure S3. Forest plot of KLF1 targets (PILON_KLF1_TARGETS_DN).

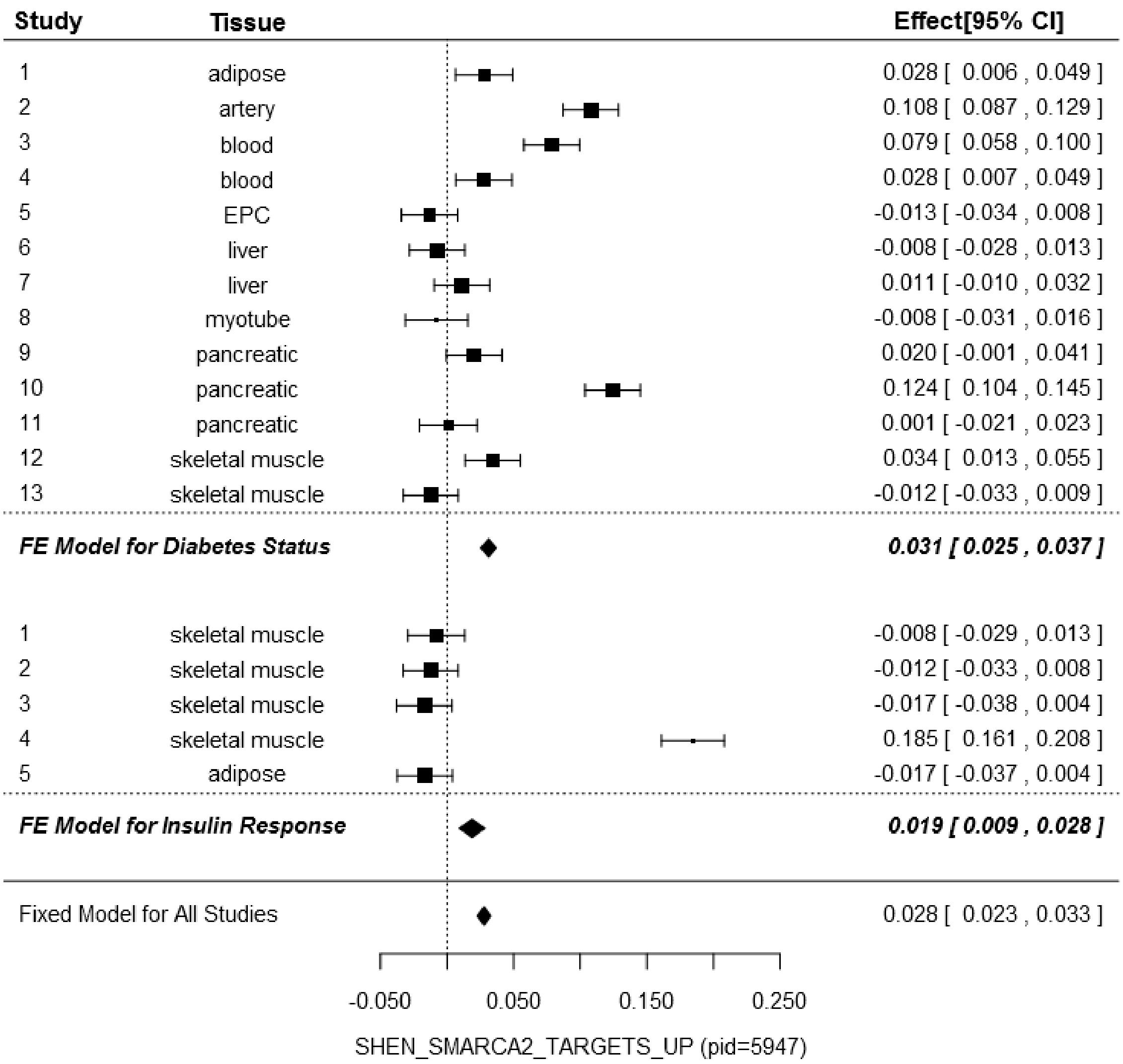

Figure S4. Forest plot of SMARCA2 targets (SHEN_SMARCA2_TARGETS_UP).

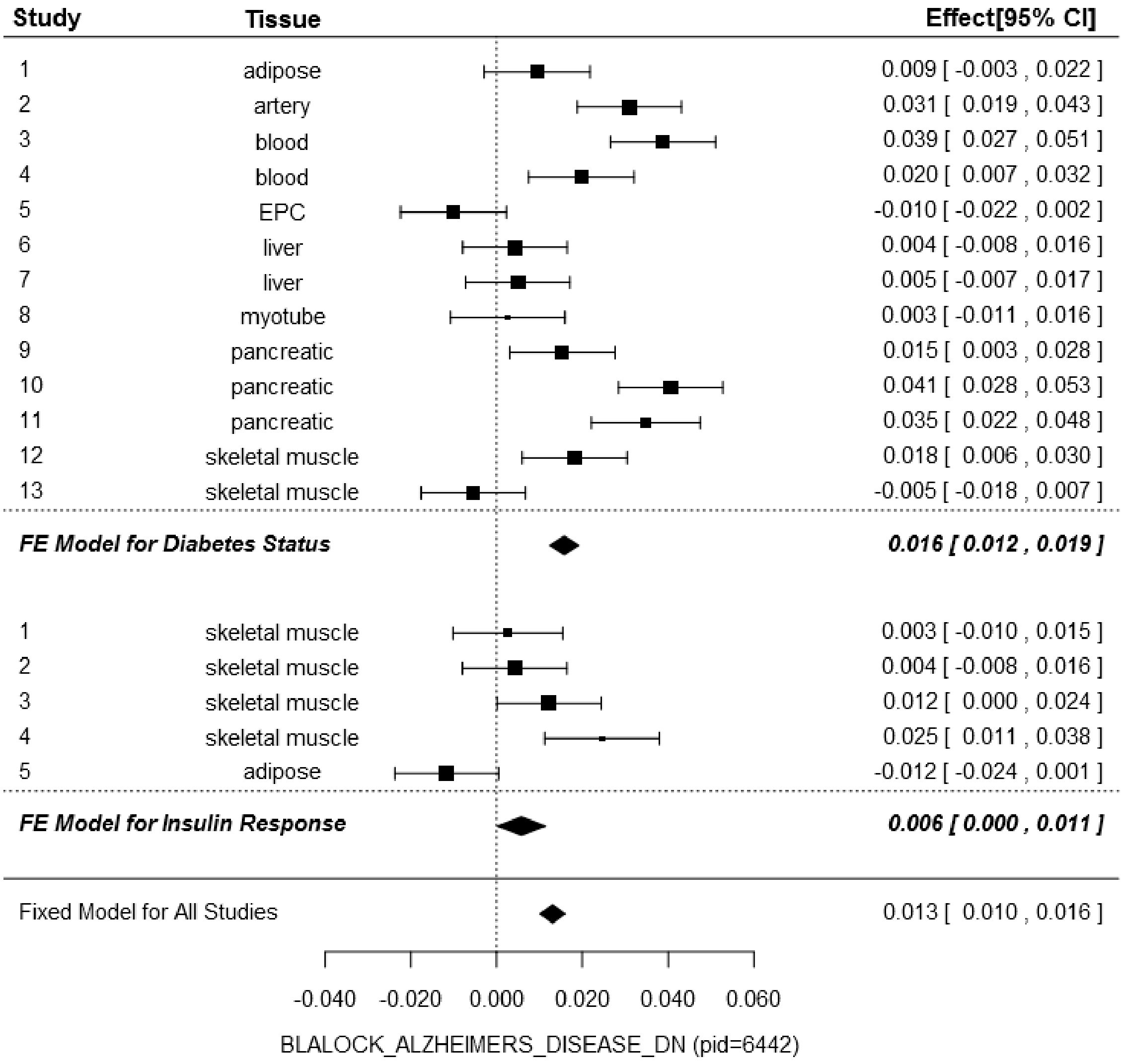

Figure S5. Forest plot of Alzheimer's disease (BLALOCK_ALZHEIMERS_DISEASE_DN).

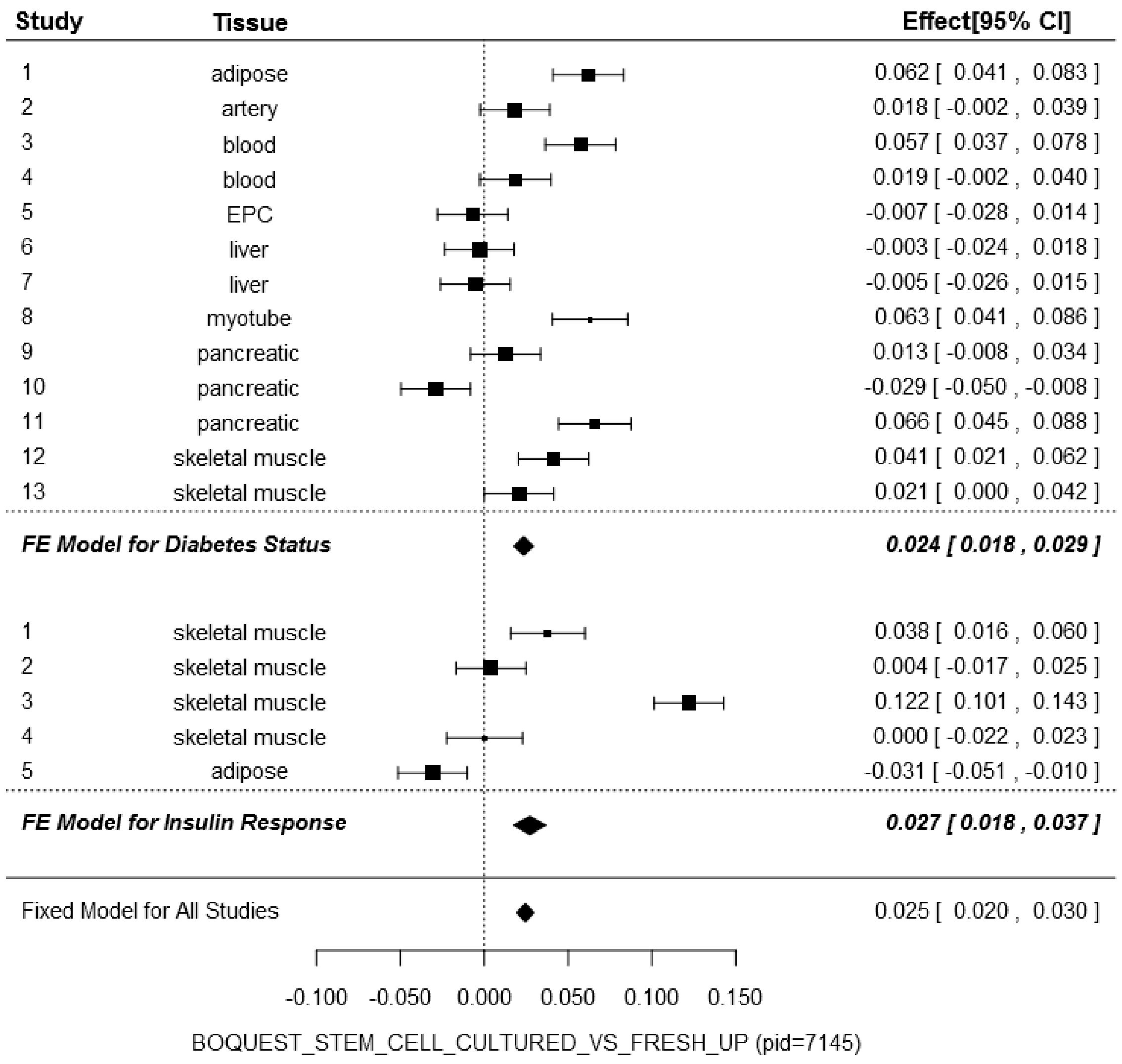

Figure S6. Forest plot of stromal stem cells (BOQUEST_STEM_CELL_CULTURED_VS_FRESH_UP).

Table S1. Description of differentially expressed genes.

Full Name	Symbol	Entrez ID	Map	Strand	Start (bp)	End (bp)
progesterone receptor membrane component 1	PGRMC1	10857	Xq22-q24	+	118370211	118378429
hydroxyacyl-CoA dehydrogenase	HADH	3033	4q22-q26	+	108910870	108956331
insulin receptor substrate 1	IRS1	3667	2q36	-	227596033	227664545
mercaptopyruvate sulfurtransferase	MPST	4357	22q13.1	+	37415683	37425863

Table S2. Description of significantly identified gene sets.

Gene Set	Name	PUBMED ID
UV response [1]	DACOSTA_UV_RESPONSE_VIA_ERCC3_DN	15608684
chronic myelogenous leukemia [2]	DIAZ_CHRONIC_MEYLOGENOUS_LEUKEMIA_UP	17252012
KLF1 targets [3]	PILON_KLF1_TARGETS_DN	18852285
SMARCA2 targets [4]	SHEN_SMARCA2_TARGETS_UP	19074882
Alzheimer's disease [5]	BLALOCK_ALZHEIMERS_DISEASE_DN	14769913
stromal stem cells of adipose [6]	BOQUEST_STEM_CELL_CULTURED_VS_FRESH_UP	15635089

1. http://software.broadinstitute.org/gsea/msigdb/cards/DACOSTA_UV_RESPONSE_VIA_ERCC3_DN; 2. http://software.broadinstitute.org/gsea/msigdb/cards/DIAZ_CHRONIC_MEYLOGENOUS_LEUKEMIA_UP; 3. http://software.broadinstitute.org/gsea/msigdb/cards/PILON_KLF1_TARGETS_DN; 4. http://software.broadinstitute.org/gsea/msigdb/cards/SHEN_SMARCA2_TARGETS_UP; 5. http://software.broadinstitute.org/gsea/msigdb/cards/BLALOCK_ALZHEIMERS_DISEASE_DN; 6. http://software.broadinstitute.org/gsea/msigdb/cards/BOQUEST_STEM_CELL_CULTURED_VS_FRESH_UP.

Table S3. Gene set enrichment analysis of UV response.

				**Diabetes**				
Study	GDS_ID	genes	sigGenes	effect	SE	pval	adjP	tissue
1	GDS3665	785	53	0.018	0.008	0.011	≥0.05	adipose
2	GDS3980	851	85	0.050	0.007	1.77E−10	1.00E−04	artery
3	GDS3874/GDS3875	851	82	0.045	0.008	8.69E−09	3.00E−04	blood
4	GDS3963	805	58	0.022	0.008	0.002	≥0.05	blood
5	GDS3656	806	28	−0.015	0.008	0.979	≥0.05	EPC
6	GDS3876	851	50	0.009	0.007	0.099	≥0.05	liver
7	GDS3883	852	50	0.009	0.007	0.107	>0.05	liver
8	GDS3681	852	44	0.002	0.007	0.374	≥0.05	myotube
9	GDS3782	852	49	0.007	0.008	0.160	≥0.05	pancreatic
10	GDS3882	851	54	0.013	0.007	0.030	≥0.05	pancreatic
11	GDS4337	797	75	0.044	0.008	4.40E−08	0.0018	pancreatic
12	GDS3880	852	58	0.018	0.008	0.009	≥0.05	skeletal muscle
13	GDS3884	852	49	0.008	0.007	0.134	≥0.05	skeletal muscle
				Insulin response				
1	GDS157/GDS158/GDS160/ GDS161/GDS162	803	48	0.010	0.008	0.090	≥0.05	skeletal muscle
2	GDS2790/GDS2791	851	58	0.018	0.007	0.006	≥0.05	skeletal muscle
3	GDS3181	851	71	0.033	0.007	6.68E−06	≥0.05	skeletal muscle
4	GDS3715	852	121	0.092	0.007	8.33E−29	<1.00E−04	skeletal muscle
5	GDS3781/GDS3962	852	37	−0.006	0.007	0.776	≥0.05	adipose

GDS_ID: the identifier of the curated GEO dataset; genes: the number of genes in the gene set with expression measured; sigGenes: the number of significant genes (i.e., *U*-score ≤ 0.05) in the gene set; effect: estimated enrichment effect; SE: the estimated standard error of effect; pval: unadjusted *p*-value of enrichment test; adj P: adjusted *p*-value of enrichment test.

Table S4. Gene set enrichment analysis of chronic myelogenous leukemia.

Study	GDS_ID	genes	sigGenes	effect	SE	pval	adjP	tissue
				**Diabetes**				
**1**	**GDS3665**	**1347**	**106**	**0.029**	**0.006**	**8.69E−07**	**0.0307**	**adipose**
**2**	**GDS3980**	**1379**	**135**	**0.048**	**0.006**	**1.56E−15**	**<1.00E−04**	**artery**
**3**	**GDS3874/GDS3875**	**1380**	**168**	**0.071**	**0.006**	**8.22E−28**	**<1.00E−04**	**blood**
**4**	**GDS3963**	**1340**	**115**	**0.036**	**0.006**	**2.68E−09**	**1.00E−04**	**blood**
5	GDS3656	1351	47	−0.015	0.006	0.997	≥0.05	EPC
6	GDS3876	1379	81	0.009	0.006	0.052	≥0.05	liver
7	GDS3883	1380	92	0.017	0.006	0.002	≥0.05	liver
8	GDS3681	1179	53	−0.005	0.006	0.785	≥0.05	myotube
9	GDS3782	1374	88	0.013	0.006	0.010	≥0.05	pancreatic
**10**	**GDS3882**	**1379**	**128**	**0.043**	**0.006**	**6.14E−13**	**<1.00E−04**	**pancreatic**
11	GDS4337	1273	73	0.007	0.006	0.098	≥0.05	pancreatic
12	GDS3880	1380	99	0.021	0.006	0.000	≥0.05	skeletal muscle
13	GDS3884	1380	71	0.001	0.006	0.366	≥0.05	skeletal muscle
				**Insulin response**				
1	GDS157/GDS158/GDS160/GDS161/GDS162	1264	67	0.003	0.006	0.291	≥0.05	skeletal muscle
2	GDS2790/GDS2791	1382	103	0.024	0.006	1.12E−05	≥0.05	skeletal muscle
3	GDS3181	1379	93	0.017	0.006	0.001	≥0.05	skeletal muscle
**4**	**GDS3715**	**1172**	**113**	**0.046**	**0.006**	**3.08E−13**	**<1.00E−04**	**skeletal muscle**
5	GDS3781/GDS3962	1380	50	−0.013	0.006	0.992	≥0.05	adipose

Refer to the legend of Table S3 for explanation of the column titles. The bold font indicates the significant enrichment analysis with adjusted *p*-value ≤ 0.05

Table S5. Gene set enrichment analysis of KLF1 targets.

Study	GDS_ID	genes	sigGenes	effect	SE	pval	adjP	tissue
**Diabetes**								
1	GDS3665	1810	113	0.012	0.005	0.005	≥0.05	adipose
**2**	**GDS3980**	**1615**	**139**	**0.036**	**0.005**	**1.44E−11**	**<1.00E−04**	**artery**
**3**	**GDS3874/GDS3875**	**1954**	**216**	**0.060**	**0.005**	**4.89E−30**	**<1.00E−04**	**blood**
4	GDS3963	1879	126	0.017	0.005	0.000	≥0.05	blood
5	GDS3656	1855	60	−0.018	0.005	1.000	≥0.05	EPC
6	GDS3876	1615	96	0.009	0.005	0.029	≥0.05	liver
7	GDS3883	1963	117	0.009	0.005	0.020	≥0.05	liver
8	GDS3681	1203	64	0.003	0.006	0.269	≥0.05	myotube
9	GDS3782	1947	122	0.012	0.005	0.006	≥0.05	pancreatic
**10**	**GDS3882**	**1615**	**121**	**0.025**	**0.005**	**1.31E−06**	**0.0443**	**pancreatic**
11	GDS4337	1787	104	0.008	0.005	0.045	≥0.05	pancreatic
12	GDS3880	1963	139	0.020	0.005	0.000	≥0.05	skeletal muscle
13	GDS3884	1963	100	0.001	0.005	0.391	≥0.05	skeletal muscle
**Insulin response**								
1	GDS157/GDS158/GDS160 /GDS161/GDS162	1740	93	0.003	0.005	0.236	≥0.05	skeletal muscle
2	GDS2790/GDS2791	1621	100	0.012	0.005	0.011	≥0.05	skeletal muscle
3	GDS3181	1615	110	0.018	0.005	0.000	≥0.05	skeletal muscle
**4**	**GDS3715**	**1194**	**103**	**0.036**	**0.006**	**3.44E−09**	**1.00E−04**	**skeletal muscle**
5	GDS3781/GDS3962	1963	81	−0.008	0.005	0.962	≥0.05	adipose

Refer to the legend of Table S3 for explanation of the column titles. The bold font indicates the significant enrichment analysis with adjusted *p*-value ≤ 0.05.

Table S6. Gene set enrichment analysis of SMARCA2 targets.

Study	GDS_ID	genes	sigGenes	effect	SE	pval	adjP	tissue
**Diabetes**								
1	GDS3665	398	31	0.028	0.011	0.006	≥0.05	adipose
**2**	**GDS3980**	**424**	**67**	**0.108**	**0.011**	**5.09E−18**	**<1.00E−04**	**artery**
**3**	**GDS3874/GDS3875**	**424**	**55**	**0.079**	**0.011**	**5.92E−11**	**<1.00E−04**	**blood**
4	GDS3963	412	32	0.028	0.011	0.005	≥0.05	blood
5	GDS3656	406	15	−0.013	0.011	0.868	≥0.05	EPC
6	GDS3876	424	18	−0.008	0.011	0.722	≥0.05	liver
7	GDS3883	424	26	0.011	0.011	0.121	≥0.05	liver
8	GDS3681	332	14	−0.008	0.012	0.698	≥0.05	myotube
9	GDS3782	422	30	0.020	0.011	0.026	≥0.05	pancreatic
**10**	**GDS3882**	**424**	**74**	**0.124**	**0.011**	**2.20E−22**	**<1.00E-04**	**pancreatic**
11	GDS4337	391	20	0.001	0.011	0.402	≥0.05	pancreatic
12	GDS3880	424	36	0.034	0.011	0.001	≥0.05	skeletal muscle
13	GDS3884	424	16	−0.012	0.011	0.856	≥0.05	skeletal muscle
**Insulin response**								
1	GDS157/GDS158/GDS160/GDS161/GDS162	404	17	−0.008	0.011	0.735	≥0.05	skeletal muscle
2	GDS2790/GDS2791	424	16	−0.012	0.011	0.859	≥0.05	skeletal muscle
3	GDS3181	424	14	−0.017	0.011	0.942	≥0.05	skeletal muscle
**4**	**GDS3715**	**328**	**77**	**0.185**	**0.012**	**6.15E−33**	**<1.00E−04**	**skeletal muscle**
5	GDS3781/GDS3962	424	14	−0.017	0.011	0.936	≥0.05	adipose

Refer to the legend of Table S3 for explanation of the column titles. The bold font indicates the significant enrichment analysis with adjusted *p*-value ≤ 0.05.

Table S7. Gene set enrichment analysis of Alzheimer's disease.

Study	GDS_ID	genes	sigGenes	effect	SE	pval	adjP	tissue
**Diabetes**								
1	GDS3665	1195	71	0.009	0.006	0.056	≥0.05	adipose
**2**	**GDS3980**	**1235**	**100**	**0.031**	**0.006**	**3.40E−07**	**0.0127**	**artery**
**3**	**GDS3874/GDS3875**	**1236**	**111**	**0.039**	**0.006**	**1.36E−09**	**1.00E−04**	**blood**
4	GDS3963	1205	84	0.020	0.006	0.001	≥0.05	blood
5	GDS3656	1202	48	−0.010	0.006	0.948	≥0.05	EPC
6	GDS3876	1235	67	0.004	0.006	0.212	≥0.05	liver
7	GDS3883	1236	68	0.005	0.006	0.187	≥0.05	liver
8	GDS3681	1025	54	0.003	0.007	0.310	≥0.05	myotube
9	GDS3782	1225	81	0.015	0.006	0.006	≥0.05	pancreatic
**10**	**GDS3882**	**1235**	**112**	**0.041**	**0.006**	**9.47E−11**	**<1.00E−04**	**pancreatic**
**11**	**GDS4337**	**1131**	**96**	**0.035**	**0.006**	**1.08E−07**	**0.0038**	**pancreatic**
12	GDS3880	1236	85	0.018	0.006	0.002	≥0.05	skeletal muscle
13	GDS3884	1236	55	−0.005	0.006	0.799	≥0.05	skeletal muscle
Insulin response								
1	GDS157/GDS158/GDS160 /GDS161/GDS162	1115	59	0.003	0.007	0.305	≥0.05	skeletal muscle
2	GDS2790/GDS2791	1235	67	0.004	0.006	0.216	≥0.05	skeletal muscle
3	GDS3181	1235	77	0.012	0.006	0.018	≥0.05	skeletal muscle
4	GDS3715	1018	76	0.025	0.007	0.000	≥0.05	skeletal muscle
5	GDS3781/GDS3962	1236	47	−0.012	0.006	0.973	≥0.05	adipose

Refer to the legend of Table S3 for explanation of the column titles. The bold font indicates the significant enrichment analysis with adjusted *p*-value ≤ 0.05.

Table S8. Gene set enrichment analysis of stromal stem cells.

Study	GDS_ID	genes	sigGenes	effect	SE	pval	adjP	tissue
**Diabetes**								
**1**	**GDS3665**	**410**	**46**	**0.062**	**0.011**	**9.49E−08**	**0.0033**	**adipose**
2	GDS3980	424	29	0.018	0.011	0.035	≥0.05	artery
**3**	**GDS3874/GDS3875**	**424**	**46**	**0.057**	**0.011**	**4.79E−07**	**0.0201**	**blood**
4	GDS3963	408	28	0.019	0.011	0.036	≥0.05	blood
5	GDS3656	417	18	−0.007	0.011	0.695	≥0.05	EPC
6	GDS3876	424	20	−0.003	0.011	0.550	≥0.05	liver
7	GDS3883	424	19	−0.005	0.011	0.643	≥0.05	liver
**8**	**GDS3681**	**362**	**41**	**0.063**	**0.011**	**2.48E−07**	**0.008**	**myotube**
9	GDS3782	424	27	0.013	0.011	0.094	≥0.05	pancreatic
10	GDS3882	424	9	−0.029	0.011	0.998	≥0.05	pancreatic
**11**	**GDS4337**	**396**	**46**	**0.066**	**0.011**	**3.54E−08**	**0.0016**	**pancreatic**
12	GDS3880	424	39	0.041	0.011	0.000	≥0.05	skeletal muscle
13	GDS3884	424	30	0.021	0.011	0.022	≥0.05	skeletal muscle
Insulin response								
1	GDS157/GDS158/GDS160 /GDS161/GDS162	374	33	0.038	0.011	0.001	≥0.05	skeletal muscle
2	GDS2790/GDS2791	424	23	0.004	0.011	0.295	≥0.05	skeletal muscle
**3**	**GDS3181**	**424**	**73**	**0.122**	**0.011**	**9.87E−22**	**<1.00E−04**	**skeletal muscle**
4	GDS3715	357	18	0.000	0.012	0.425	≥0.05	skeletal muscle
5	GDS3781/GDS3962	424	8	−0.031	0.011	0.999	≥0.05	adipose

Refer to the legend of Table S3 for explanation of the column titles. The bold font indicates the significant enrichment analysis with adjusted *p*-value ≤ 0.05.

Table S9. Significant correlation of gene expression association.

Study1_ID	Study2_ID	Study1_GDS	Study2_GDS	Rho	*p*-Value
2	3	GDS3980	GDS3874/GDS3875	0.036	5.98E−04
1	8	GDS3665	GDS3681	0.038	4.62E−04
3	12	GDS3874/GDS3875	GDS3880	0.040	1.21E−04
2	12	GDS3980	GDS3880	0.041	1.07E−04
12	13	GDS3880	GDS3884	0.041	8.05E−05
1	3	GDS3665	GDS3874/GDS3875	0.047	1.72E−05
2	7	GDS3980	GDS3883	0.055	2.25E−07
1	11	GDS3665	GDS4337	0.057	5.59E−07
3	4	GDS3874/GDS3875	GDS3963	0.065	6.65E−09
2	10	GDS3980	GDS3882	0.074	7.07E−12

Table S10. Significant independence of gene expression association.

Study I ID	Study II ID	Study I Data	Study II Data	Chi-square	*p*-Value
5	12	GDS3656	GDS3880	12.156	4.89E−04
4	5	GDS3963	GDS3656	13.493	2.39E−04
3	6	GDS3874/GDS3875	GDS3876	15.959	6.47E−05
3	7	GDS3874/GDS3875	GDS3883	17.817	2.43E−05
3	13	GDS3874/GDS3875	GDS3884	18.738	1.50E−05
5	11	GDS3656	GDS4337	18.872	1.40E−05
5	9	GDS3656	GDS3782	21.134	4.28E−06
3	8	GDS3874/GDS3875	GDS3681	22.227	2.42E−06
3	10	GDS3874/GDS3875	GDS3882	22.367	2.25E−06
3	5	GDS3874/GDS3875	GDS3656	37.894	7.47E−10

Table S11. Significant correlation of pathway expression association.

Study I ID	Study II ID	Study I Data	Study II Data	Rho	*p*-Value
1	13	GDS3665	GDS3884	0.032	6.22E−04
6	12	GDS3876	GDS3880	0.035	1.67E−04
1	11	GDS3665	GDS4337	0.036	1.57E−04
9	12	GDS3782	GDS3880	0.037	7.64E−05
3	6	GDS3874/GDS3875	GDS3876	0.040	2.29E−05
7	11	GDS3883	GDS4337	0.040	2.17E−05
11	12	GDS4337	GDS3880	0.041	1.52E−05
7	13	GDS3883	GDS3884	0.043	7.59E−06
4	6	GDS3963	GDS3876	0.045	2.79E−06
4	7	GDS3963	GDS3883	0.056	6.92E−09
3	9	GDS3874/GDS3875	GDS3782	0.056	5.71E−09
7	9	GDS3883	GDS3782	0.056	5.40E−09
3	11	GDS3874/GDS3875	GDS4337	0.060	4.55E−10
2	6	GDS3980	GDS3876	0.061	2.71E−10
1	8	GDS3665	GDS3681	0.064	4.17E−11
2	11	GDS3980	GDS4337	0.066	1.50E−11

5	13	GDS3656	GDS3884	0.066	1.29E−11
8	9	GDS3681	GDS3782	0.074	3.02E−14
3	10	GDS3874/GDS3875	GDS3882	0.075	1.38E−14
7	8	GDS3883	GDS3681	0.077	4.90E−15
1	7	GDS3665	GDS3883	0.080	3.43E−16
7	12	GDS3883	GDS3880	0.080	1.91E−16
7	10	GDS3883	GDS3882	0.081	6.75E−17
4	10	GDS3963	GDS3882	0.084	5.32E−18
2	8	GDS3980	GDS3681	0.085	4.61E−18
6	10	GDS3876	GDS3882	0.085	2.04E−18
1	4	GDS3665	GDS3963	0.092	3.22E−21
3	7	GDS3874/GDS3875	GDS3883	0.097	3.38E−23
11	13	GDS4337	GDS3884	0.099	5.49E−24
4	12	GDS3963	GDS3880	0.099	5.29E−24
10	12	GDS3882	GDS3880	0.104	2.60E−26
1	12	GDS3665	GDS3880	0.111	1.14E−29
8	13	GDS3681	GDS3884	0.115	8.36E−32
9	11	GDS3782	GDS4337	0.116	1.92E−32
1	2	GDS3665	GDS3980	0.119	4.40E−34
2	4	GDS3980	GDS3963	0.130	2.32E−40
2	7	GDS3980	GDS3883	0.132	1.71E−41
5	8	GDS3656	GDS3681	0.144	1.37E−48
1	3	GDS3665	GDS3874/GDS3875	0.146	4.16E−50
3	12	GDS3874/GDS3875	GDS3880	0.146	2.67E−50
1	10	GDS3665	GDS3882	0.157	3.52E−58
2	12	GDS3980	GDS3880	0.164	2.78E−63
2	3	GDS3980	GDS3874/GDS3875	0.167	9.10E-66
8	11	GDS3681	GDS4337	0.195	4.78E−88
2	10	GDS3980	GDS3882	0.211	9.20E−104
3	4	GDS3874/GDS3875	GDS3963	0.241	5.17E−136
